# Atovaquone exerts its anticancer effect by inhibiting Na^+^/K^+^-ATPase ion transport in canine cancer cells

**DOI:** 10.14202/vetworld.2023.1185-1192

**Published:** 2023-06-04

**Authors:** Yousef Alharbi

**Affiliations:** Department of Veterinary Medicine, College of Agriculture and Veterinary Medicine, Qassim University, Qassim, Saudi Arabia

**Keywords:** atovaquone, electrophysiology, Na^+^/K^+^-ATPase, oxidative phosphorylation, oxidative stress, plumbagin

## Abstract

**Background and Aim:**

New anticancer drugs are being developed to avoid the toxicity and chemoresistance of the currently available drugs. The Food and Drug Administration-approved anti-malarial drug atovaquone is known to act as a selective oxidative phosphorylation inhibitor in the mitochondria by competing with CO Q10 (mitochondrial complex II and III). This study aimed to investigate the effect of atovaquone by examining the Na^+^/K^+^-ATPase (NKA) activity in various canine cell lines.

**Materials and Methods:**

Canine cell lines were treated with various concentrations (2.5, 5, 10, 15, and 20 μM) of atovaquone for 24, 48, and 72 h. Human cell lines were used as a control to validate the canine cancer cell lines. The activities of the drugs against the cancer cell lines were measured using the 3-(4,5-dimethylthiazol-2-yl)-2,5-diphenyl-2H-tetrazolium bromideassay. The cell metabolic activity was determined by measuring the activities of the nicotinamide adenine dinucleotide phosphate-dependent cellular oxidoreductase enzymes. The NKA activity was measured using the single-cell patch clamping assay.

**Results:**

Atovaquone-induced apoptosis by elevating the concentration of reactive oxygen species (ROS) in the tumor cells, leading to cell death. Treatment of canine cancer cells with N-acetylcysteine (ROS inhibitor) reduced the activity of the drug. Furthermore, atovaquone inhibited more than 45% of the NKA ion current.

**Conclusion:**

This study demonstrated effects of atovaquone against canine cancer cell lines. The data may prove beneficial in repurposing the drug as a new anticancer agent in canine clinical trials, which might aid in fighting human cancer.

## Introduction

Developing novel anticancer agents is an ongoing process due to the lack of an effective drug to eradicate the disease. Most patients treated with anticancer drugs present with relatively good outcomes; however, some develop chemoresistance [1–4]. In most cases, recurrent cancer does not respond to chemotherapy, and the tumors become aggressive and malignant [5–8]. In addition, the currently available anticancer drugs have high toxicity and extreme side effects; consequently, they cannot be used on cancer patients with other severe diseases. Therefore, developing anticancer agents with low toxicity and chemotherapy resistance is essential. Studies suggest that repurposing currently available drugs to avoid their toxicity and reduce the duration of clinical trial studies might prove beneficial [9–13]. The Food and Drug Administration (FDA) in the United States (US) has approved the use of atovaquone for treating malaria. Several studies have shown that atovaquone can inhibit human cancer cell lines by inhibiting mitochondrial complexes I and II, leading to a massive increase in reactive oxygen species (ROS) [14–17]. Furthermore, this drug is cheaper than the currently available chemotherapeutical agents.

Atovaquone can kill cancer cells at a half maximal inhibitory concentration (IC_50_) of 10–30 nM [14, 16, 17], which is significantly lower than the IC_50_ of the standard chemotherapeutic drug cisplatin (20–50 μM) [18, 19]. Furthermore, atovaquone was reported to inhibit Na^+^/K^+^-ATPase (NKA) activity in human cancer cell lines and negatively influenced the proliferation of both human and canine cancer cell lines [16]. Thus, targeting the NKA channel with this drug will decrease the survival rates of cancer cells.

This study aimed to investigate the use of atovaquone as an anticancer agent by evaluating the NKA activity in various canine cell lines.

## Materials and Methods

### Ethical approval

This study (*in vitro*) does not require ethical approval.

### Study period and location

The study was conducted from November to April 2021, in the Technical Laboratory at the Department of Veterinary Medicine, College of Agriculture and Veterinary Medicine, Qassim University, Saudi Arabia.

### Cell culture and antibodies

Human (OV-2008, SKOV-3, and ECC1) and canine (Payton, CTAC-3, CMT-27, and Denny) cancer cell lines were obtained from the University of Wisconsin-Madison College of Veterinary Medicine (US). Human cell lines were used as a control to validate canine cell lines. The cells were maintained in Dulbecco’s modified Eagle’s medium supplemented with 10% fetal calf serum and incubated at 37°C in a 5% carbon dioxide (CO_2_) atmosphere.

The primary antibodies targeting cleaved caspase 3, superoxide dismutase (SOD), phosphorylated-focal adhesion kinase (p-FAK), and β-actin were purchased from cell signaling technologies (Danvers MA, USA). Horseradish peroxidase-conjugated goat anti-rabbit secondary antibodies and sheep anti-mouse secondary antibodies were purchased from Jackson ImmunoResearch (West Grove PA, USA). All other reagents were obtained from ThermoFisher (Waltham, MA, USA).

### Cell viability assay

The activities of the drugs against cancer cell lines were measured using the (3-[4,5-dimethylthiazol-2-yl]-2,5 diphenyl tetrazolium bromide (MTT) assay. The cell metabolic activity was determined by measuring the activities of the nicotinamide adenine dinucleotide phosphate H-dependent cellular oxidoreductase enzymes. The human and canine cancer cells (1 × 10^4^/well) were plated in a 96-well plate and incubated overnight in a humidified incubator at 37°C and 5% CO_2_ atmosphere. The cells were treated with different concentrations (2.5, 5, 10, 15, and 20 μM) of atovaquone for 72 h. The control cells were treated with dimethyl sulfoxide (DMSO). After incubation, 20 μL of MTT reagent (50 μg/mL final concentration/well) was added to each well, and the plates were incubated at 37°C for another 4 h. The media and MTT were discarded, and 100 μL of DMSO was added to each well. The plate was placed on a horizontal shaker for 8 min to mix the reagents and dissolve the formazan crystal. The optical densities of the wells were measured at 560 nm on a microplate reader machine. Whole-cell patch clamping was performed on a single Payton cultured cell using a micropipette to evaluate the NKA activity after treatment with atovaquone. The bath solution consisted of calcium chloride and barium chloride (BaCl_2_) to inhibit the K^+^ channels and cadmium chloride (CdCl_2_), nickel (II) chloride (NiCl_2_), and Nifedipine to inhibit the Ca^++^ channels.

### Western blot

Cancer cells (6 × 10^6^) were plated onto 10 cm tissue culture dishes and incubated overnight. The cells were treated with atovaquone or DMSO at different times (24, 48, and 72 h). Subsequently, they were lysed with radioimmunoprecipitation assay buffer containing 0.1% sodium dodecyl sulfate, 25 mM Tris-hydrochloride pH 7.6, 150 mM sodium chloride, 0.5% sodium deoxycholate, and 15 Triton X-100 and a protease inhibitor cocktail (10× protease inhibitors in stock solution). The lysates were harvested from the culture plates, kept on ice, and then transferred to 2 mL Eppendorf tubes. The lysates were sonicated on ice for 30 s to prevent protein denaturation and centrifuged at 4°C for 30 min. The supernatants were transferred to two new 2 mL Eppendorf tubes and stored at −15°C until further analysis. The protein concentration of each lysate was measured using the BCA assay (ThermoFisher, IL, USA). The lysate (25 μg/lane) was typically loaded onto each sodium dodecyl sulfate (SDS)-polyacrylamide gel electrophoresis gel lane. Depending on the molecular weight of the protein, 7.5% or 12% polyacrylamide gels were run at a constant voltage of 150 mV for 1 h in running buffer (25 mM Tris, 119 mM Glycine, and 1 g/L SDS; pH 7.5). Proteins from the gel were transferred to a polyvinylidene fluoride (PVDF) membrane using the sandwich transfer method in transfer buffer (25 mM Tris and 119 mM Glycine; pH 7.5) at 250 mA for 1 h on ice. The PVDF membranes were blocked in 20 mL tris-buffered saline with 0.1% Tween 20 detergent (TBST) buffer (5 mM Tris containing 0.015 M sodium chloride and 500 μl/L of Tween 20, pH 7.5) containing 5% milk powder at 22 ± 2°C for 1 h. Then, the membranes were incubated with the primary antibody (1:1000 dilution) in 1× TBST buffer containing 5% dry milk at 4°C overnight. The membranes were washed 3 times (15 min each) with 20 mL of 1× TBST buffer and incubated with horseradish peroxidase-conjugated secondary antibody (typically, 1:30,000 dilution) in TBST buffer containing 5% milk powder. After incubation for 1 h at 22 ± 2°C, the membranes were washed 3 times with TBST buffer. The antibody was detected using the WestPico, Dura, or WestFemto detection reagents (ThermoFisher); the bands were detected using an X-ray film.

### Electrophysiology

Whole-cell patch clamping was used to measure the ion current in the pump. The NKA ion currents were isolated using an inhibitor to block the Ca^2+^Na^+^/K^+^ and Ca^2+^/K^+^ channels. K^+^ currents were inhibited by removing K^+^ from the internal solution and adding BaCl_2_ to the external solution. Na^+^/Ca^2+^ exchange ion currents were inhibited by adding NiCl_2_ to the external solution and removing Ca^2+^ from the internal and external solutions. Ca^2+^ currents were eliminated by adding CdCl_2_ and NiCl_2_ to the external solution and removing Ca^2+^ from the external solution. Na^+^/Ca^2+^ exchange currents were inhibited by adding NiCl_2_ to the external solution and removing Ca^2+^ from the internal and external solutions. The experiment involved measuring currents that ranged from +50 mV as a depolarization to −160 mV as a hyperpolarization.

The holding membrane potential was applied in step-wise from −160 mV, wherein +50 mV was applied to stimulate membrane depolarization, and the outward current of the pump was activated (3Na^+^ exit the cell) to maintain the membrane potential. However, the application of −160 mV stimulated the membrane hyperpolarization and activated the NKA inward currents (2K^+^ enter the cell).

The cells were exposed to 10 μM of atovaquone, following which inward and outward currents during time course protocol were measured using the ramp and voltage step program. The current through the NKA was mathematically determined by calculating the difference before and after treatment with atovaquone. The time courses of the effects of the drugs on the NKA current at all voltage pulses were determined.

### Immunofluorescence of the NKA-alpha subunit in the Payton cells

Payton cells were labeled with an anti-NKA-alpha subunit antibody and tagged with a fluorescent secondary antibody. In addition, the cells were stained with the nuclear marker 4′,6-diamidino-2-phenylindole (DAPI). Quantification of the immunofluorescence showed the location of the NKA-alpha subunit on the cell membrane.

### Statistical analysis

The results from all experiments were plotted in GraphPad Prism program 7 software (https://www.graphstats.net/). Statistical analysis was conducted using the features available through this software package. Typically, we used the one-way analysis of variance test to determine statistical significance and calculate the p-values.

## Results

### Atovaquone inhibits the proliferation of human and canine cancer cells

**Figure-1:**
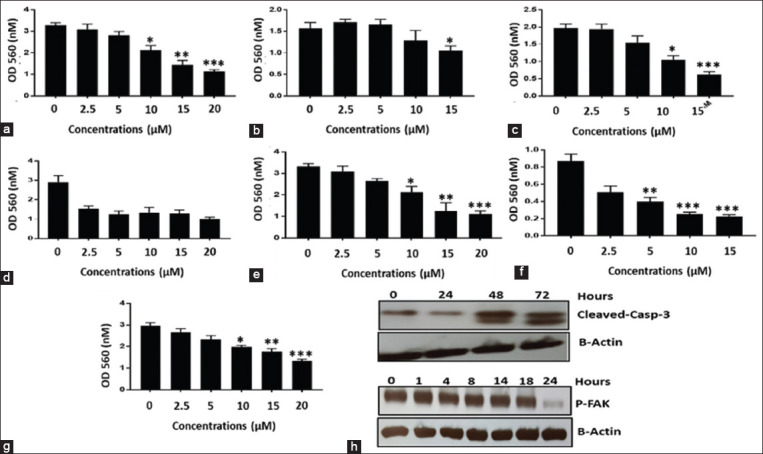
Atovaquone inhibits the proliferation of different human and animal cancer cell lines. The effect of Atovaquone on the viability of human (OV-2008, SKOV-3, and ECC1 a–c, respectively) and canine cancer cell lines (Payton, CTAC, CM-27, and Denny d-g, respectively) was determined by MTT assays is shown in Figure-1. MTT assay demonstrates the inhibitory activity of Atovaquone on the viability of the cancer cell lines. Each bar is an average of three biological replicates with significant levels at; *p = 0.012–0.08, **p = 0.00274, and ***p = 0.00062. Western blot analysis of payton cells treated with Atovaquone (10 μM) for different time points. Cleaved caspase and p-FAK were shown by through western blot and β-actin as loading control.

Atovaquone exhibited cytotoxic effects on the human (Figures-1a-c) and canine (Figures-1d-g) cancer cell lines. The drug inhibited proliferation at IC_50_ (10–20 μM; Figures-1a-g) by causing cell death through apoptosis. Pretreatment with 10 μM of atovaquone at the three different time points (24, 48, and 72 h) increased the amount of cleaved Caspase-3 compared to that in the DMSO-treated (control) cells. Western blotting revealed a decrease in the phosphorylation of FAK, especially after 24 h of treatment.

The MTT assay revealed the inhibitory effect of atovaquone on the viability of human (Figures-1a-c) and canine (Figures-1d-g) cancer cell lines. Each bar in the figure represents an average of three biological replicates with significant levels at: *p = 0.012–0.08, **p = 0.00274, and ***p = 0.00062. Payton cells were given various doses of atovaquone (10 M) and were then subjected to a western blot analysis. Western blotting was used to demonstrate cleaved caspase and p-FAK, with -actin serving as the loading control.

### Reactive oxygen species mediate the activity of atovaquone

**Figure-2:**
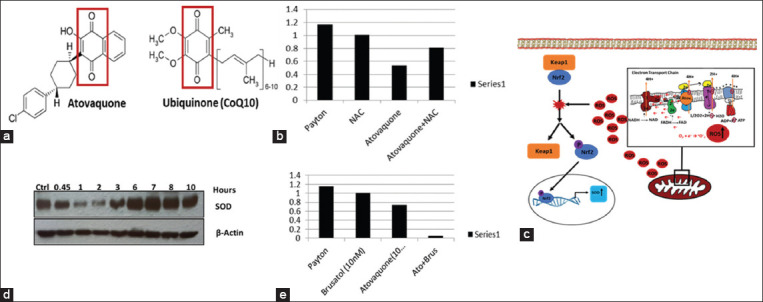
Reactive oxygen species (ROS) mediate the activity of atovaquone. (a) The structure of Atovaquone and ubiquinone (CoQ10) showed the similarity between them in the benzene ring. (b) MTT data for Payton cells were treated with dimethyl sulfoxide (DMSO) (control), NAC, NAC+ Atovaquone for 72 h. (c) Cartoon showed the ROS affect in the Nrif-2 expression as survival mechanism. (d) Western blot for payton cells treated with 10 μM of Atovaquobe for different time points, and (e) MTT data for payton cells treated with Brusatol, Atovaquone, Brusatol + Atovaquone and DMSO (control) for 72 h.

Atovaquone is like ubiquinone in terms of the quinone ring (CoQ10) (Figure-2a). The Payton cells were pretreated with 1 mM N-acetyl cysteine (NCA) for 15 min, which was then washed away using phosphate-buffered saline buffer; 10 μM of atovaquone was applied to the cells or 72 h. The MTT data showed that pre-incubation of the cells with NCA decreased the activity of atovaquone when compared to that in the cells treated cells with atovaquone alone (Figure-2b). The results showed that increasing the ROS resulted in increased nuclear factor erythroid 2–related factor 2 (Nrf-2) (transcription factor) protein expression and eventually increased the antioxidant markers such as SOD (Figure-2c). Payton cells were treated with 10 μM of atovaquone at different time points (0.45, 1, 2, 3, 6, 7, 8, and 10 h). Western blotting revealed a decrease in the expression of SOD during the first 2 h, which was then followed by a sharp increase in expression (Figure-2d). The increase served as a survival mechanism to reduce the level of ROS. To prove this hypothesis, the Payton cell lines were treated with 10 μM of atovaquone alone or in combination with 10 nM of the Nrf-2 inhibitor brusatol. Inhibition of Nrf-2 resulted in the death of cancer cells (Figure-2e).

### Effects of ROS-mediated atovaquone on NKA inhibition

**Figure-3:**
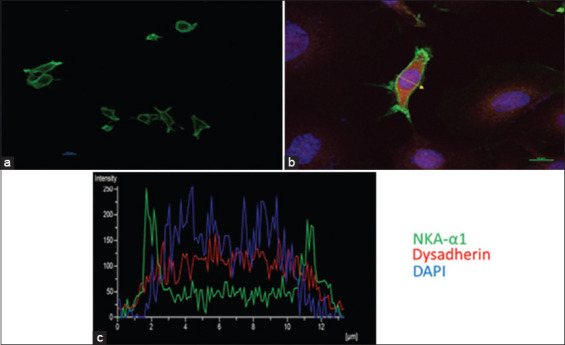
(a and b) Immunofluroscence of NKA in payton cell. Flureoscence microscopy was used to show the expression of the NKA in the payton cell membrane. Payton cell was labeled with anti NKA-α subunit antibody and fluorescence tagged with secondary antibody. (b) The cell was also stained with DAPI which gave blue color for nuclei. The image shows expression of NKA on the cell membrane of the Payton cell. (a and b) Distribution of NKA observed on the cell surface as shown in the fluroscence image (c) as well as the fluroscence quantification plots.

Immunofluorescence microscopy was used to measure the expression of the NKA-α subunit in the Payton cells. The NKA-α subunit was highly expressed in the cell membranes (green fluorescence), while the DAPI (blue fluorescence) was expressed in the cellular nuclei (Figures-3a and b). Fluorescence microscopy was used to observe the expression of the NKA in the Payton cell membrane. The distribution of NKA was also observed using fluorescence quantification plots (Figure-3c).

### The activity of the NKA ion channel in the atovaquone-treated payton cells

**Figure-4:**
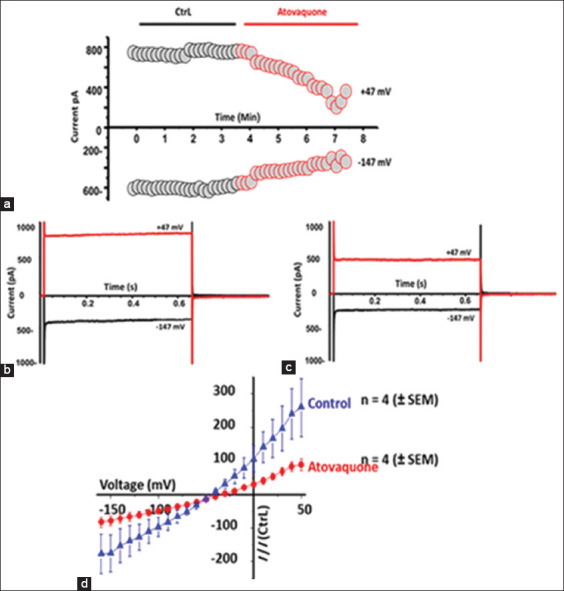
The activity of NKA ion channel mediated-Atovaquone treated cells. (a) The time course experiment shows the ion current in the control (black trace) and Atovaquone (10 μM, red trace) in Payton-treated cells. The current was measured in the two different voltages +47 mV as outward current and −147 mV as inward current starting from the holding potential of −60 mV. (b) Twenty one different voltages in steps matter starting from +47 mV to −147 mV. The plot on the left shows the measurement of the ion current in the control cell. The outward current at +47 mV was ~900 pA and the inward current at −147mV was ~400 pA. In the right plot, the ion current was decreased after the cell was treated with 10 μM of atovaquone. The outward current was reduced to ~500 pA (red trace) and the inward current was reduced to ~230 pA (Black trace). (c) The average normalized current-voltage (I-V) curve of total of four cells. (d) The control (blue trace) and after the application of atovaquone (10 μM, red trace) are shown. The results indicate a 43% inhibition of current in payton cells treated with atovaquone (n = 4; p = 0.00225).

The outward and inward currents over time are shown in Figure-4a; the black trace is the control, and the red trace is the current after applying 10 μM of atovaquone. The application of the drug in the bath solution inhibited the outward and inward currents (Figure-4a). In a single Payton cell, voltage measurements were made at 21 different voltages in steps ranging from +47 mV to -147 mV. The current was applied in only two different voltage steps: +47 mV for the outward current and −147 mV for the inward current (red and black traces, respectively; Figures-4b and c). The outward and inward currents were recorded before (Figure-4b) and after (Figure-4c) applying 10 μM of the drug. At both +47 mV and -147 mV, the outward and inward currents were reduced. The average of the normalized current-voltage (I-V) curve from four cells was plotted. The NKA activity was decreased in the atovaquone-treated cells (red trace; Figure-4d). The percentage of inhibition was about 45% of the current when compared to that in the control cells.

The time course experiment showing the ion current in the control and atovaquone-treated Payton cells. The current was measured at two different voltages: +47 mV for outward current and −147 mV for inward current, starting from a holding potential of −60 mV. Twenty-one different voltages in a step-wise manner starting from +47mV to −147 mV. The plot on the left shows the ion current measurement in the control cell. The outward current at +47 mV was ~900 pA, and the inward current at −147 mV was ~400 pA. The plot on the right shows a decrease in the ion current after treating the cells with 10 μM of atovaquone. The outward current was reduced to ~500 pA, while the inward current was reduced to ~230 pA. The average normalized current-voltage (I–V) curve of a total of four cells. The control and after the application of atovaquone are shown. The results indicated a 43% inhibition in payton cells treated with atovaquone (n = 4; p = 0.00225).

### Effect of atovaquone in chemoresistant cells

**Figure-5:**
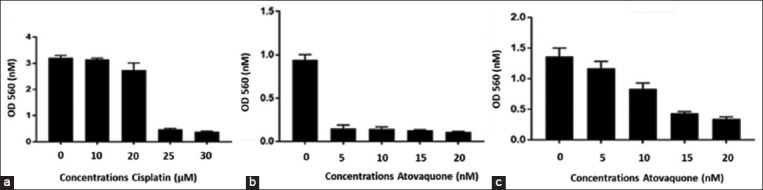
The activity of atovaquone in chemotherapy resistance cells. (3-[4,5-Dimethylthiazol-2-yl]-2,5 diphenyl tetrazolium bromide showed C13 cells treated with different concentrations of atovaquone for 72 h (a). (3-[4,5-Dimethylthiazol-2-yl]-2,5 diphenyl tetrazolium bromide showed that cisplatin resistance C13 cells were treated with different concentrations of cisplatin (b) or Atovaquone (c) for 72 h.

The treatment of C13 cells with different concentrations of atovaquone inhibited cell proliferation (Figure-5a). A cisplatin-resistant Payton cell line was developed to evaluate the effect of atovaquone. No significant inhibition was detected in cells treated with 10 μM and 20 μM of cisplatin (Figure-5b); however, the proliferation was inhibited after treatment with 5 μM of atovaquone (Figure-5c). Cisplatin-resistant C13 cells were exposed to various doses of cisplatin or atovaquone for 72 h.

## Discussion

This study aimed to investigate the effect of atovaquone by determining the NKA activity in various human and canine cell lines. Atovaquone is an FDA-approved anti-malarial agent that inhibits electron transport (ETC) in parasitic infections and cancer cells [20–22]. The anticancer effects of this drug in human and animal cancer cell lines have been demonstrated previously [16, 17, 23–25].

Using a canine cancer cell line as a model for human cancer, atovaquone caused apoptosis, which might be tested in a clinical study as an anticancer. Focal adhesion kinase protein is an important biomarker for the growth, proliferation, survival, and migration of cancer cells; it is highly expressed in tumor tissues but not in normal tissues [26]. The activity of atovaquone was mainly ascribed to its potential as an anti-oxidative stress agent [16, 17, 23–25]. Low levels of ROS after treatment with atovaquone are the major cause of cancer cell death [27], and inhibition of ROS with a ROS inhibitor (NAC) reduced the activity of the tested drug [28]. Thus, activating the survival mechanism by increasing the expression of antioxidant proteins and reducing the ROS level is essential [27–29]. Superoxide dismutase, one of the most common antioxidants, was reported to be expressed after treatment with atovaquone [30–32]. We believe that the ring in the atovaquone structure competes with CoQ10 and inhibits the mithochondrial electron transport leading to the leaking of electron radicals and eventually increases the ROS level [16]. We showed that ROS is a major cause of apoptosis cell death in human cancer cells [16].

Furthermore, studies have shown that the inhibition of the ETC complexes (I and II) not only increases the level of ROS, but also causes a dramatic reduction in the production of ATP [17, 24, 33]. NKA is known to consume more than 40% of the ATP in the cell [34, 35]. Therefore, a reduction in ATP production caused by inhibiting the ETS complexes in the mitochondria will directly affect the NKA activity [36, 37]. The previous studies suggest that the increase in ROS caused by the inhibition of the ETC complexes can induce apoptosis and oxidize the NKA-α/β subunits [38, 39]. The oxidation of these subunits can inhibit the NKA activity and promote its degradation [25, 38, 40–46]. As the survival mechanism, we believe that increasing the ROS will cause increased nuclear factor erythroid 2–related factor 2 (Nrf-2) (transcription factor) protein expression and eventually increase the antioxidant markers such as SOD (Figure-2c).

In this study, the activity of NKA was measured using the single-cell patch clamping assay. The activity of the pump was reduced after treatment with atovaquone. Na^+^/K^+^-ATPase is an important factor for cell survival and is highly expressed in cancer cells compared to normal cells [47–49]. Therefore, the targeting of NKA would prove beneficial in reducing the survival rate, especially in chemoresistant cancer cells [50–52]. In this study, reductions in proliferation were observed in cisplatin-resistant cancer cells treated with atovaquone.

The main limitation of this study is the lack of implementation in patients; however, the future studies are warranted to evaluate the long-term effects of various doses of atovaquone in human subjects.

## Conclusion

This study showed that the anti-malarial drug atovaquone could be used as a second-line treatment in cancer patients. The findings of the study may help in the repurposing of the drug as a novel anticancer agent in canine clinical trials and aid in our fight against cancer in humans.

## Authors’ Contribution

YA: Proposed the idea, planned the study, executed the treatments, and drafted and revised the manuscript. The author read, reviewed, and approved the final manuscript.
